# Effects on Personal Factors Through Flipped Learning and Gamification as Combined Methodologies in Secondary Education

**DOI:** 10.3389/fpsyg.2020.01103

**Published:** 2020-06-12

**Authors:** Adrián Segura-Robles, Arturo Fuentes-Cabrera, María Elena Parra-González, Jesús López-Belmonte

**Affiliations:** ^1^Department of Research Methods and Diagnosis in Education, University of Granada, Granada, Spain; ^2^Department of Didactics and School Organization, University of Granada, Granada, Spain

**Keywords:** autonomy, motivation, Physical Education, flipped learning, gamification

## Abstract

**Purpose:**

This study aims to analyze the effects of a flipped and gamified program on the autonomy, competence, relation with others, satisfaction/enjoyment, intrinsic and extrinsic motivation, and boredom of students of Physical Education.

**Method:**

The study used a control group and an experimental group to compare pretest and posttest data in both of them. Instruments used were the Basic Psychological Needs in Exercise Scale, Sport Motivation Scale, and Sport Satisfaction Instrument, all of them validated in academic literature.

**Results:**

On one hand, data indicated that autonomy has been increased with the application of these teaching methodologies. On the other hand, students’ satisfaction, enjoyment, and intrinsic motivation have improved based on the interaction with gamification and flipped learning. Finally, with all dimensions, it seems that academic performance has been improved, although not in a significative way.

**Discussion/Conclusion:**

Results of the study provide to educational researchers valuable information for a better understanding of how flipped learning and gamification influence personal performance of Physical Education students.

## Introduction

The intrinsic technological nature of the current era is promoting continuous changes in people’s daily actions (Maldonado et al., 2019). Especially, in the field of education, technology is reaching a leading role (Area et al., 2016). This has led to the emergence of new ways of teaching and learning content from an innovative perspective, in which students assume a greater role (Li et al., 2019). Educational innovation in the new millennium has driven the emergence and constant development of training activities focused on student participation (Pereira et al., 2019). All this derives in the promulgation of the concept of active methodology as a new way of transmitting and generating a knowledge shared and elaborated by the students themselves, guided by the teacher for an optimal achievement of the objectives and consolidation of the contents (Kerrigan, 2018).

This new direction taken by the teaching practice has meant an increase in the motivation and attitude of the students (Álvarez-Rodríguez et al., 2019) due to the new opportunities and means of learning that the students have within their reach (Mat et al., 2019), bringing today’s education closer to the singularities of a digital society (Nikolopoulou et al., 2019). This has encouraged not only the deployment of new ways of imparting the contents but also the appearance of new spaces and times for the instructional process (Nogueira et al., 2018), what is known as ubiquity (Cabero and Barroso, 2018).

To adapt today’s education to the new demands of students developed in a time of great technological stimulation, the innovative actions of teachers, to improve the quality of training and student satisfaction, have resulted in the projection of methodological approaches. Among them are gamification and its variants such as escape room, and flipped learning, as a combination between the face-to-face and virtuality of learning moments (Parra-González et al., 2020). Specifically, in the subject of Physical Education, innovative practices are proliferating to impart the contents from a new approach in order to encourage the attitude of students on their way to effective and motivating learning (Quintero et al., 2018; Kang and Kang, 2019; Rodríguez et al., 2019; Wyant and Baek, 2019).

Gamification is one of the active methodologies that has reached a great penetration in the learning spaces of our day (Sánchez et al., 2020). This formative approach is based on the game and the transformation of formal contexts into playful learning spaces (Hanus and Fox, 2015; González et al., 2016). Despite its expansion in current teaching practice, the use of games for content delivery has been developing since the 1960s (Malone and Lepper, 1987). This game-centered approach aims to facilitate the student’s effort to achieve the learning objectives and adapt to the demands, needs, and interests of the collective (Oliva, 2017; Parra-González et al., 2019). The games used have evolved over time, from the most classic of childhood to those with a large digital load that generate immersion in a virtual reality (Contreras-Espinosa, 2016). And its potential has been verified in different educational stages, positioning it as a suitable methodology for any age range (Giannakos, 2013; Dib and Adamo-Villani, 2014; Su and Cheng, 2015).

With gamification, students prepare their own knowledge structures supported by academic indicators benefited such as motivation (Pisabarro and Vivaracho, 2018; Groening and Binnewies, 2019), attitude (Lee et al., 2013; Pérez-Manzano and Almela-Baeza, 2018), interest, autonomy (Xi and Hamari, 2019), commitment (Chu and Hung, 2015), dedication (Simões et al., 2013; Wang, 2015), attraction (Area and González, 2015), collaboration (Perrotta et al., 2013), interactions between agents (teacher-students and content) (Parra-González and Segura-Robles, 2019), and problem-solving capacity (Kapp, 2012) in activities to develop.

An essential aspect of gamification is the incentive that the student receives in any progress or step that is being achieved, which has a positive impact on the psychosocial indicators, previously exposed and that are directly connected with the performance obtained by the students (Mekler et al., 2017). The recent literature on gamified practices in Physical Education reflects the potential of this teaching and learning methodology, contributing to the improvement of various academic indicators, such as motivation, involvement, and student satisfaction (Mora-González et al., 2020). The escape room is conceived as a training modality based on the gamification of the student’s learning environment. This didactic approach is based on the resolution of challenges and problems raised that give rise to various instructional situations where students have to put their knowledge into practice (Agreda et al., 2019).

This methodological innovation is based on a game, challenge, or problem that students have to solve both autonomously (self-management of knowledge) and collaboratively (shared knowledge management), encouraging the participation of students in a problematic situation of a real nature or invented by the teacher (Wynn and Okie, 2017). In every escape room, students are locked in classrooms or different spaces where they have to perform different tasks, activities, challenges, tests, and riddles, among others, to obtain the “key” in a certain time that will allow them to leave the place (López-Pernas et al., 2019).

The expert literature on the state of the matter reflects that the development of training practices through escape room promotes the improvement of various academic indicators already mentioned previously by gamification. Specifically, this gamified practice improves motivation (Borrego et al., 2017), activation, participation (Sierra and Fernández Sánchez, 2019), satisfaction, attitude, and attraction of students for the learning actions to be carried out (Pérez et al., 2019) to assimilate and reinforce the contents (Eukel et al., 2017).

All these improvements produced have a direct and positive impact on student grades and, consequently, student performance (Hursen and Bass, 2019). The latter is enhanced as a result of this innovative practice of transmitting, reinforcing, and consolidating knowledge developed by the student himself in a collaborative learning experience experienced and experienced in the first person (Kinio et al., 2019).

In the field of Physical Education, these innovative practices of a gamified nature are already being carried out. Recent research reveals proposals and recommendations to efficiently perform an escape room in the classrooms to impart the contents of that subject. Also, experts in this line of study offer guidance, resources, and ideas for optimal development by teachers less experienced in the use of this training methodology (Flores, 2019; Segura and Parra, 2019).

Due to the high rate of sedentary lifestyle and obesity in the youngest population (Talarico and Janssen, 2018), it is necessary to increase the time of physical activity during the class time of the Physical Education subject. Students are in as long as possible on the move One solution to reduce the time of explanations of the contents, activities, tasks, and games to be performed is flipped learning. This innovative methodology is presented as a mix between face-to-face and digital training (Mengual et al., 2020).

This innovation of a hybrid nature implies a greater use of class time and greater activity of students because the explanations have been made prior to the face-to-face session and digitally in other contexts (Bergmann and Sams, 2012). The teacher’s role is focused on generating audiovisual content so that students can view them on any mobile device with an Internet connection (López et al., 2019a). In this way, class time is devoted to deepening the didactic content and solving the students’ doubts since the phase of explanation and assimilation of contents has been carried out outside the classroom (Bognar et al., 2019). This promotes an alteration and inversion of learning moments. First, the student visualizes the content at home or in any place suitable for learning and in the classroom, the assimilated in the digital environment is reinforced and implemented (Long et al., 2017).

Flipped learning is presented in impact literature as a didactic approach that has achieved relevant popularity and effectiveness at different levels, stages, and educational contexts (He et al., 2016; López et al., 2019b; Zainuddin et al., 2019). All this is reflected in various studies that demonstrate how the application of this mixed approach to learning improves motivation (Tse et al., 2019), teamwork (Kwon and Woo, 2017), attitude (Lee et al., 2018), participation and activation (Chyr et al., 2017), autonomy (González and Carrillo, 2016), commitment (Huang et al., 2018), and interactions between the agents involved and with the contents (Hwang et al., 2015). All this has a positive impact on students’ grades and performance (Karabulut et al., 2018; Nortvig et al., 2018; Sola et al., 2019).

In particular, the application of flipped learning in Physical Education has shown encouraging results as reflected in the reported studies (Hinojo et al., 2018, 2020), placing this innovative approach as a methodological alternative to impart the own contents of Physical Education and, in the same way, increase intrinsic factors of the learning process such as motivation, autonomy, problem solving, the use of class time, the interaction between teacher–student, student–student, and student–content, and the deepening of content and ratings.

In short, different recent high-impact researches show the potentialities of the instructional approaches previously presented (Luong, 2019; Østerlie and Kjelaas, 2019; Quintas et al., 2020; Sargent and Casey, 2020). The novelty that this study presents concerning the existing literature focuses on the simultaneous integration of both training methodologies for the approach of contents linked to the subject of Physical Education. Despite the scientific volume that covers these teaching and learning methodologies separately in the field of education in general, the interest aroused in this work focuses on the combination of both methodological innovations in the field of physical activity and sport. Therefore, research is required to promote a change in the point of view of professionals in this educational sector, as well as the development of new training experiences adapted to the new times (Cañabate et al., 2019).

## Materials and Methods

### Research Design and Data Analysis

This research has been carried out through experimental and pre–post design based on the quantitative design as indicated by experts (Hernández et al., 2014; Pérez-Escoda, 2018). This type of design has already demonstrated its effectiveness as a tool to know the effects of different tools or methodologies in the educational field (Juliá-Hurtado and Baena-Extremera, 2018; Epelde-Larrañaga et al., 2020). The objective of pre–post studies is to observe over time and to check the influence of different variables on the study population. In traditional pre–post studies, differences are only observed in one study group (Gravetter and Forzano, 2009). In order to give the results more robustness, a control group is added to the study to which no methodologies are applied. The objective is to provide the results with greater validity.

The students were divided into two groups, which we named experimental and control groups. There was a pretest and a posttest analysis in each group. The intervention on the experimental group consisted of teaching through the methodology and technique of flipped learning and escape room. In contrast, the control group follows a traditional methodology, without the use of any specific methodology.

All statistics have been carried out with the Statistical Package for the Social Sciences (SPSS) v25 program. Before carrying out the different analysis, the normality of the sample is checked, using the Kolmogorov–Smirnov test, and obtaining significant results, so that non-parametric statistics is used to perform inferential analysis. To test effect size, we use Cohen d. Effect size is a quantitative measure of the magnitude of the experimenter effect. The larger the effect size, the stronger the relationship between two variables (Harlow et al., 2016; Lenhard and Lenhard, 2016).

### Participants

The participants who took part in this research were 64 students enrolled at third grade of secondary school. Other studies of a certain impact show that the size of the sample in this kind of researches does not condition the performance of these experiments (Rodríguez, 2011).

The selection of the sample was carried out through an intentional sampling due to the ease of access to the students. They are enrolled in an educational secondary school in the Autonomous City of Ceuta (Spain). This research came out as a need that one of the researchers, who also work there as a teacher, detected that something needed to be done to improve a situation.

Specifically, the students were selected from the third year of Secondary Education (*n* = 64; mean age = 15 years; *SD* = 1.62). The configuration of the two groups on which the experimentation was carried out is specified in Table 1.

**TABLE 1:** Study groups by sex.

Groups	Boys *n* (%)	Girls *n* (%)	Total *n* (%)
Experimental group	17 (53.12)	15 (46.87)	32 (50)
Control group	11 (34.75)	21 (65.62)	32 (50)
Subtotal	28 (43.75)	36 (56.25)	64 (100)

### Instruments

The instruments used to carry out this research were four scales. The first one was the Basic Psychological Needs in Exercise Scale; the version used in this research was the one validated and translated into Spanish (Moreno et al., 2008). The dimensions used and to be analyzed were autonomy, competence, and relation with others (for example, I feel like I can communicate openly with my classmates). It had a Likert-type scale from 1 (totally disagree) to 5 (totally agree).

The second scale, which was used, was Sport Motivation Scale, which was adapted to Physical Education as well (Granero-Gallegos et al., 2014), used to analyze extrinsic and intrinsic motivation (for example, for the pleasure of living stimulating experiences), also with a Likert-type scale from 1 (totally disagree) to 5 (totally agree).

The third scale used was the Sport Satisfaction Instrument. The one used was the Spanish version also adapted to Physical Education (Baena-Extremera et al., 2012) to measure the satisfaction/enjoyment and boredom (for example, I usually find Physical Education in English interesting), also with a Likert-type scale from 1 (totally disagree) to 5 (totally agree). In addition, the last scale was used only at the posttest moment to measure the academic achievement. The decision about which factors were going to be analyzed was taken due to the dimensions of the questionnaire, the factors that can be influenced by the implementation of active methodologies on teaching and learning process, and both of these aspects were followed up after the scientific literature revision which was done.

### Procedure

**FIGURE 1:**
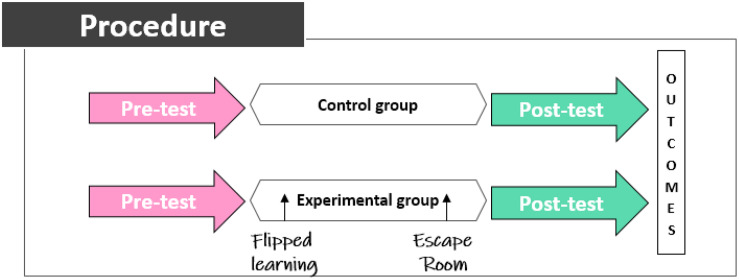
Experimentation procedure.

In relation to the procedure, pretests were administered to both control and experimental groups. Then, a teaching methodology based on flipped classroom and escape room was used with the experimental group. Afterward, posttests were applied on both groups to analyze the outcomes (Figure 1). Time between pretests and posttests was 5 weeks.

### Intervention

A didactic unit consisting of eight sessions on contents related to physical condition and health was carried out to work the physical qualities such as strength, endurance, and speed. With the control group, the sessions were carried out in a traditional way. The teacher was in charge of designing the physical tasks and activities to be carried out by the student. The role assumed by the teacher in this group was purely expository and face-to-face. The teacher focused on explaining the activities of each session in the corresponding installation, always in person, and the student limited himself to carrying out the tasks without taking the lead or choosing the actions to be carried out. The training actions focused on general fitness circuits using training materials, as well as cooperative motor games. On the contrary, in the experimental group, the teacher presented audiovisual materials on a content management platform so that the students could visualize them before going to the face-to-face session. Once the students went to the class, they already had a knowledge base and information about the activities and challenges to carry out. In this group, the teacher’s role is more passive. The student acquires all the prominence since he is the main agent who has to carry out each of the tests, challenges, and enigmas of the designed escape room. The tests, challenges performed by the students in the escape room had a physical component with the purpose of working on the physical qualities mentioned above. In addition, students could dress up and characterize based on the theme of the escape room. Therefore, the teacher grants the students of the experimental group autonomy and freedom to investigate, explore, and collaborate to pass all the tests and solve the final puzzle, thus including all the elements used during the experimentation.

## Results

Before carrying out the different descriptive and inferential analyses, the reliability of the instruments used through Cronbach’s alpha is checked. All the results obtained show satisfactory results for each of the dimensions (Bonett and Wright, 2015).

In addition, the omega index (ω) is calculated, which, unlike the alpha coefficient, works with the factor loads, being a test less biased than the classical alpha (Salazar Vargas and Serpa Barrientos, 2017). In the same way, the results show acceptable values, between 0.70 and 0.90 (Table 2; Campo-Arias and Oviedo, 2008).

**TABLE 2:** Reliability and validity indices.

	Alpha (α)	Omega (ω)
Autonomy	0.87	0.89
Competence	0.90	0.88
Relation with others	0.81	0.83
Satisfaction/enjoyment	0.89	0.85
Extrinsic motivation	0.82	0.83
Intrinsic motivation	0.80	0.78
Boredom	0.84	0.81

Table 3 shows the values obtained in the pre–post tests performed for the control group and the experimental group being similar. The highest values in the control group were obtained for competition (4.61 ± 0.13) and the lowest for extrinsic motivation (2.21 ± 0.83). Similarly, the highest values in the pretest control group were obtained for satisfaction (3.29 ± 1.02). It is in the values of the subsequent test of the experimental group that the data seem to change significantly, so that, following the main objective of the study, inferential analyses are performed on pre–post values in the control group and the experimental group to prove it.

**TABLE 3:** Mean (M) and standard deviation (SD) of the results for the pretest and posttest.

Traditional Learning (Control group)		
	Pretest	Posttest
	
	Mean (SD)	Mean (SD)
Autonomy	4.09 (0.25)	4.15 (0.11)
Competence	4.10 (0.27)	4.61 (0.13)
Relation with others	3.53 (0.12)	3.49 (0.32)
Satisfaction/enjoyment	3.29 (1.02)	3.12 (0.32)
Extrinsic motivation	2.50 (0.73)	2.21 (0.83)
Intrinsic motivation	2.41 (0.51)	2.51 (0.60)
Boredom	3.31 (0.25)	3.50 (0.42)
Learning achievement		4.71 (1.91)

**Gamified–Flipped Learning (Experimental group)**		

	**Pretest**	**Posttest**
	
	**Mean (SD)**	**Mean (SD)**
Autonomy	3.99 (0.14)	4.81 (0.21)
Competence	3.82 (0.37)	4.50 (0.33)
Relation with others	4.01 (0.24)	4.91 (0.12)
Satisfaction/enjoyment	3.62 (0.71)	4.71 (0.47)
Extrinsic motivation	2.51 (0.51)	2.91 (0.61)
Intrinsic motivation	2.11 (0.42)	3.60 (0.51)
Boredom	3.20 (0.15)	1.10 (0.52)
Learning achievement		4.90 (1.01)

Table 4 shows the results obtained after the analysis of the values obtained in the posttest in learning achievement dimension. This dimension is calculated using the evaluation obtained by the students at the end of classes, so it is only considered as a posttest result. Values do not show significant differences (*Z* = −1.125; *p* = 0.071). These differences, despite being unique, do not show great strength (*d* = 0.11). In this case, flipped learning obtained a higher mean rank (*MR* = 60.82) value than traditional learning (*MR* = 40.23).

**TABLE 4:** Mann–Whitney *U* test for learning achievement.

		Mean Rank	U	Z	*p*	d*
**Posttest**	**Traditional**	51.13	112.000	−1.125	0.071	–
	**Gamified–Flipped Learning**	55.12				

*The effect size of the statistical analyses was determined using Cohen’s d (d_cohen_) (Cohen, 1988).

Similarly, results are analyzed in the control group (pre–post) in each of the dimensions analyzed. Table 5 does not show significant differences between pre and post values in most dimensions. It is only Intrinsic motivation (*Z* = −2,288, *p* = 0.55, *d* = 0.42), in this case, effect size can be considered medium.

**TABLE 5:** Wilcoxon test for dimensions in pre–post in traditional learning.

Dimensions	Groups	Mean Rank	*Z*	*P*	*d*
Autonomy	Pretest	40.11	1,181	0.052	–
	Posttest	42.55			
Competence	Pretest	27.17	2,224	0.055	–
	Posttest	29.09			
Relation with others	Pretest	53.35	1,347	0.261	–
	Posttest	56.19			
Satisfaction/enjoyment	Pretest	33.12	2,389	0.055	–
	Posttest	27.11			
Extrinsic motivation	Pretest	65.17	−1,288	0.071	–
	Posttest	61.35			
Intrinsic motivation	Pretest	21.13	3,436	0.002*	0.42
	Posttest	32.17			
Boredom	Pretest	38.12	−2,017	0.264*	–
	Posttest	33.94			

*Significant difference.

In Table 6, Wilcoxon signed-rank test shows that flipped and gamified intervention in the experimental group had a statistically significant change in five of seven dimensions analyzed. Autonomy has improved positively, obtaining results of *Z* = 1,781, *p* = 0.03. Although these differences are significant, they show a small effect size (*d* = 0.22). In the same way, Relation with others (*Z* = 1,047, *p* = 0.02, *d* = 0.13), Satisfaction/enjoyment (*Z* = 2,389, *p* = 0.05, *d* = 0.29), intrinsic motivation (*Z* = 2,288, *p* = 0.03, *d* = 0.31), and Boredom (*Z* = −3,017, *p* = 0.02, *d* = 0.33) show significant differences too. Effects sizes to all of them can be considered small. Only Competence (*Z* = 2,224, *p* = 0.60) and Extrinsic motivation (*Z* = −2,288, *p* = 0.55) do not show significant differences.

**TABLE 6:** Wilcoxon test for dimensions in pre–post in flipped and gamified learning.

Dimensions	Groups	Mean Rank	*Z*	*p*	*d*
Autonomy	Pretest	41.10	1,781	0.003*	0.22
	Posttest	53.60			
Competence	Pretest	31.07	2,224	0.060	–
	Posttest	33.10			
Relation with others	Pretest	61.32	1,047	0.002*	0.13
	Posttest	69.27			
Satisfaction/enjoyment	Pretest	23.12	2,389	0.005*	0.29
	Posttest	42.98			
Extrinsic motivation	Pretest	73.97	−2,288	0.055	–
	Posttest	60.10			
Intrinsic motivation	Pretest	21.13	2,288	0.003*	0.31
	Posttest	41.87			
Boredom	Pretest	49.01	−3,017	0.002*	0.33
	Posttest	32.10			

*Significant difference.

## Discussion

The present study shows the influence of techniques related to new teaching models, in learning outcomes, and in different variables related to it. Current teaching must at some time be a process of constant change in teaching practice and in the daily memory of students, with constant transformations (Maldonado et al., 2019). For this, the use of active methodologies that lead to the achievement of objectives is essential (Kerrigan, 2018), as well as the use of new ways to impart in terms of spaces and time they are related (Nogueira et al., 2018).

In this experimentation, progress in learning has been compared, referring to different variables, of two groups, which have served as control and experimental groups.

The values studied in both groups have been measured before the experiment. Subsequently, a measurement of these values has been made, obtaining interesting and specific results with the application of the new methodologies.

From the study, it can be extracted that values such as autonomy have been increased with the application of these teaching models coinciding with Parra-González et al. (2020), which is to be expected especially in the application of inverted learning as a tool for the acquisition of knowledge. In addition, social relations have been increased as a result of the implementation of new methods, as indicated by other studies (Rodríguez et al., 2019).

It should be noted that the increase in satisfaction and enjoyment that students have obtained based on the interaction with gamification and flipped learning, which corroborate the results and coincide with what has been indicated in other investigations (Area and González, 2015) and in other studies related to psychosocial factors (Mekler et al., 2017). Of course, and in accordance with what has been indicated in previous research (Pisabarro and Vivaracho, 2018), there is also an increase in the intrinsic motivation of students, which implies a better acceptance of teaching and a greater predisposition to learning, eliminating glimpses of boredom in students, which entails and connects, directly, with a better performance obtained by students, as in other cases of implementation of these methodologies (Groening and Binnewies, 2019).

However, in the experimentation, there are no significant changes in variables such as student competence, as well as in extrinsic motivation, which is not consistent with that reported by other studies and authors (Mora-González et al., 2020), without influencing the future of the student (Hinojo et al., 2020) and without having an important significance for the final result.

The prospect of this study focuses on making the scientific community aware of the potential of the combination of active methodologies, both face-to-face and digital, in the teaching and learning process in the field of Physical Education, with the purpose to raise awareness among the teaching group of the benefits reported after its application.

The main limitation of this research focuses on the nature of the sample, which is situated in a single context with certain peculiarities at a social and geographical level. This projects that the results achieved here are taken with caution as the replication of this research in other contexts may vary the findings presented here.

Therefore, as a future line of study, this study is intended to be carried out in different contexts in order to verify and consolidate the potential and advantages of this methodological combination. It can be concluded that both flipped learning for content teaching and gamification to achieve the objectives are methodologies that improve student learning, as well as multiple factors associated with the education of students in current classrooms and autonomy, relationship with others, enjoyment, intrinsic motivation, and boredom. The appearance of these teaching methods should not be renounced in a changing world that has its greatest challenge in teaching.

### Permission to Reuse and Copyright

Figures, tables, and images will be published under a Creative Commons CC-BY license, and permission must be obtained for use of copyrighted material from other sources (including re-published/adapted/modified/partial figures and images from the internet). It is the responsibility of the authors to acquire the licenses, to follow any citation instructions requested by third-party rights holders, and cover any supplementary charges.

## Data Availability Statement

The datasets generated for this study are available on request to the corresponding author.

## Ethics Statement

Ethical review and approval was not required for the study on human participants in accordance with the local legislation and institutional requirements. Written informed consent to participate in this study was provided by the participants’ tutors.

## Author Contributions

AS-R and MP-G conceived the hypothesis of this study, analyzed the data, and performed the data interpretation of statistical analysis. AF-C and JL-B participated in data collection and wrote the manuscript with the most significant input. All authors contributed, read, and approved the final manuscript.

## Conflict of Interest

The authors declare that the research was conducted in the absence of any commercial or financial relationships that could be construed as a potential conflict of interest. The handling Editor AE declared a shared affiliation, though no other collaboration with the authors at the time of review.
